# Identification of novel biomarkers, shared molecular signatures and immune cell infiltration in heart and kidney failure by transcriptomics

**DOI:** 10.3389/fimmu.2024.1456083

**Published:** 2024-09-16

**Authors:** Qingqing Long, Xinlong Zhang, Fangyuan Ren, Xinyu Wu, Ze-Mu Wang

**Affiliations:** ^1^ Division of Nephrology and Clinical Immunology, Medical Faculty, Rheinisch-Westfälische Technische Hochschule (RWTH) Aachen University, Aachen, Germany; ^2^ Institute for Photogrammetry and Geoinformatics, University of Stuttgart, Stuttgart, Germany; ^3^ Division of Organic Chemistry - Bioorganic Chemistry, Mathematics/Natural Sciences Faculty, Koblenz University, Koblenz, Germany; ^4^ Faculty of Medicine, RWTH Aachen University, Aachen, Germany; ^5^ Department of Cardiology, The First Affiliated Hospital of Nanjing Medical University, Nanjing, Jiangsu, China

**Keywords:** heart failure, kidney failure, CDK2, CCND1, cell cycle, inflammation, immunology

## Abstract

**Introduction:**

Heart failure (HF) and kidney failure (KF) are closely related conditions that often coexist, posing a complex clinical challenge. Understanding the shared mechanisms between these two conditions is crucial for developing effective therapies.

**Methods:**

This study employed transcriptomic analysis to unveil molecular signatures and novel biomarkers for both HF and KF. A total of 2869 shared differentially expressed genes (DEGs) were identified in patients with HF and KF compared to healthy controls. Functional enrichment analysis was performed to explore the common mechanisms underlying these conditions. A protein-protein interaction (PPI) network was constructed, and machine learning algorithms, including Random Forest (RF), Support Vector Machine-Recursive Feature Elimination (SVM-RFE), and Least Absolute Shrinkage and Selection Operator (LASSO), were used to identify key signature genes. These genes were further analyzed using Gene Set Variation Analysis (GSVA) and Gene Set Enrichment Analysis (GSEA), with their diagnostic values validated in both training and validation sets. Molecular docking studies were conducted. Additionally, immune cell infiltration and correlation analyses were performed to assess the relationship between immune responses and the identified biomarkers.

**Results:**

The functional enrichment analysis indicated that the common mechanisms are associated with cellular homeostasis, cell communication, cellular replication, inflammation, and extracellular matrix (ECM) production, with the PI3K-Akt signaling pathway being notably enriched. The PPI network revealed two key protein clusters related to the cell cycle and inflammation. CDK2 and CCND1 were identified as signature genes for both HF and KF. Their diagnostic value was validated in both training and validation sets. Additionally, docking studies with CDK2 and CCND1 were performed to evaluate potential drug candidates. Immune cell infiltration and correlation analyses highlighted the immune microenvironment, and that CDK2 and CCND1 are associated with immune responses in HF and KF.

**Discussion:**

This study identifies CDK2 and CCND1 as novel biomarkers linking cell cycle regulation and inflammation in heart and kidney failure. These findings offer new insights into the molecular mechanisms of HF and KF and present potential targets for diagnosis and therapy.

## Introduction

Heart failure (HF) is a life-threatening problem which impacts over 64 million individuals globally (1). Concurrently, chronic kidney disease (CKD) represents a growing public crisis with a prevalence of 10-13% (2). The coexistence of heart and kidney failure (KF) is linked to significantly increased morbidity, mortality, and healthcare expenditures, alongside detrimental clinical outcomes (3, 4). The intricate interplay between the heart and kidneys is such that dysfunction in one organ can precipitate complications in the other. The prevalence of HF and CKD in tandem is not uncommon. This bidirectional relationship, where heart or kidney disorders can induce dysfunction in the counterpart organ, manifests acutely and chronically as cardiorenal syndrome (5).

The heart and kidney interact in both physiological and pathological manners. The heart relies on the kidney’s critical role in maintaining homeostasis, while the kidney’s function is intricately tied to adequate blood perfusion—a process governed by neurohormonal, hemodynamic, and inflammatory mechanisms, which are essential for maintaining salt-water balance and normal blood pressure (6). Dysregulation of the heart-kidney cross talk contributes to cardiovascular diseases, CKD, and other systemic dysfunctions (7). Recent insights suggest that the bidirectional pathways and feedback loops underpinning heart and renal failure are more complex than previously recognized (8). Emerging research indicates that cardiac and renal disorders share common pathways, such as inflammation, endothelial dysfunction, hemodynamic instability, neurohormonal activation, metabolic anomalies, and oxidative stress (9–12), along with frequently co-existence risk factors like hypertension, diabetes, obesity, and vascular complications (13), which pose challenges in medical therapy. However, the mechanisms underlying the interdependence of heart and kidney failure remain elusive. It is hence important to explore the diverse mechanisms involved in the progression of the both diseases.

The traditional diagnostic and evaluative methods for patients with HF and CKD, which rely on serum creatinine or Brain Natriuretic Peptide (BNP) levels, are limited as they reflect changes in only one organ at a time. Nevertheless, recent studies have reported several biomarkers that improve the assessment of kidney disorder severity and accurately indicate the risk of cardiorenal syndrome progression, such as NGAL, NAG, KIM1, and PCS, while still primarily detecting the deterioration of renal function (14–17). Consequently, further research is needed to identify shared novel biomarkers for diagnosis of the HF and CKD, as well as to elucidate the molecular and cellular physiological mechanisms associated with the both diseases, which may provide new targets for treatment.

To address current diagnostic limitations and deepen our understanding of the heart-kidney interplay, our study aims to advances our knowledge of the molecular mechanisms shared between heart and kidney failure, and also to uncover novel biomarkers for diagnostic and therapeutic targets. This research is pioneering in leveraging transcriptomic analysis within an integrated bioinformatics tools and machine learning algorithms to explore HF and KF. The findings are expected to provide new insights into these conditions, ultimately paving the way for improved management of cardiorenal syndrome.

## Materials and methods

### Study design

**Figure 1:**
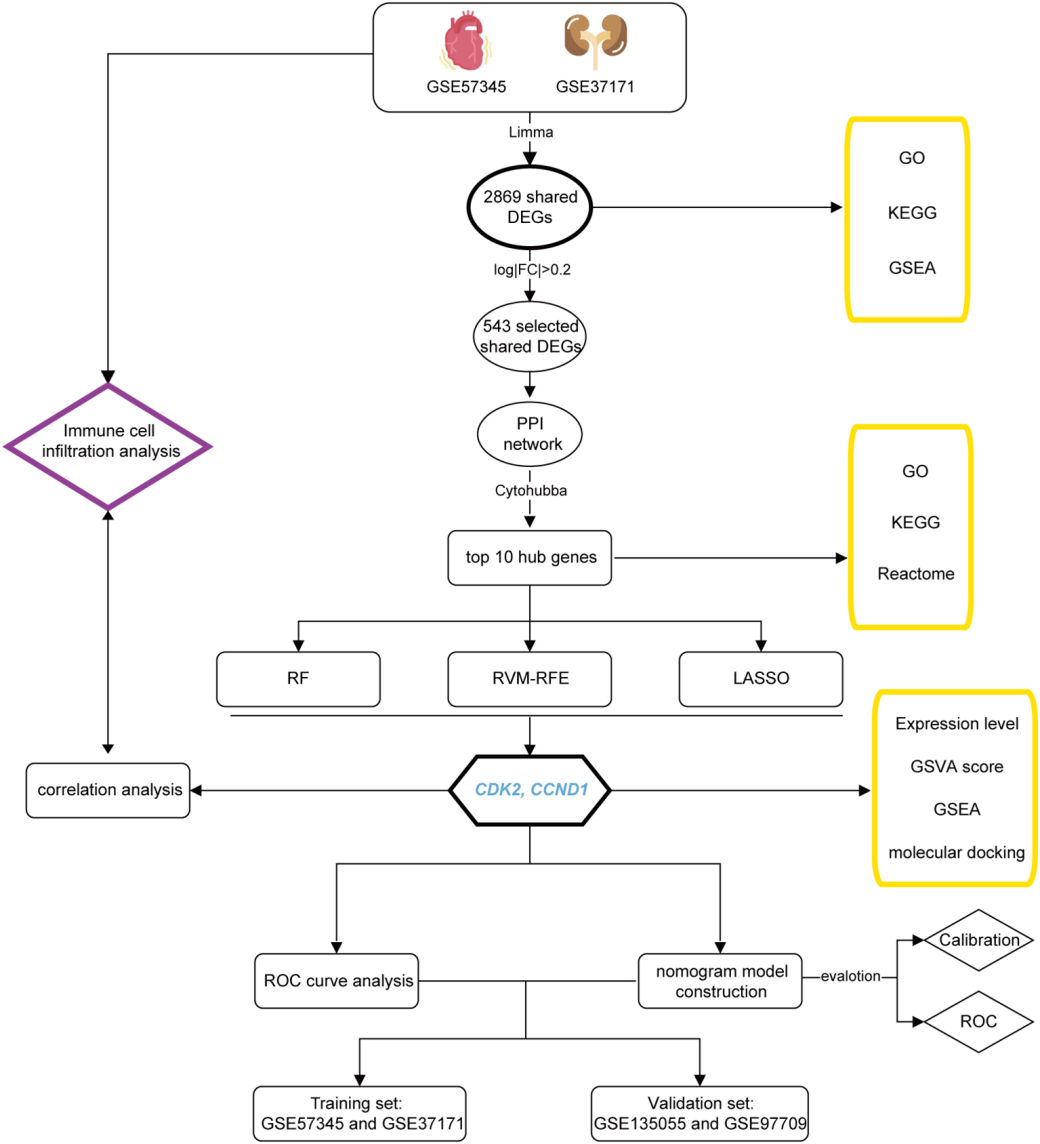
Schematic diagram of the study workflow.

The workflow of this study is depicted in **Figure 1**.

### Data collection

The keywords “heart failure” and “kidney failure” were used to search on the Gene Expression Omnibus (GEO, http://www.ncbi.nlm.nih.gov/geo/), a comprehensive public repository for genomics data (18). Two datasets, GSE57345 (which includes the gene expression profile of HF patients and healthy controls) and GSE37171 (comprising the gene expression profile of KF patients and healthy controls), were extracted from the GEO database as the training set. To validate the findings from these datasets, we acquired two additional datasets, GSE135055 (HF and controls) and GSE97709 (KF and controls), from GEO to function as our validation set. The selected datasets met specific criteria: they were derived from human subjects, utilized expression profiling by array, and each had a total sample size of at least 30, encompassing individuals with heart failure or kidney failure along with control subjects. Details regarding all datasets are listed in **Supplementary Table 1**.

### Identification of shared DEGs

The datasets were processed using the R programming language (version 4.3.2) and Bioconductor (http://bioconductor.org/biocLite.R). Initially, raw datasets underwent background correction, log2 transformation, and normalization via the ‘affy’ and ‘limma’ packages in R. Subsequently, the ‘ComBat’ algorithm within the ‘sva’ package was applied to correct for batch effects due to technical variations among the samples. To identify differentially expressed genes (DEGs) of statistical significance and biological relevance, even with modest expression difference, the ‘limma’ package was employed with a significance threshold set at an adjusted p-value <0.05 and |logFC| > 0 for GSE57345 (HF) and GSE37171 (KF), respectively, to evaluate DEGs between patients and healthy controls. Ultimately, a total of 2869 mutual upregulated and downregulated DEGs in both GSE57345 and GSE37171 were identified using Venn diagrams in the TBtools software (19).

### Functional enrichment analysis

The Gene Ontology (GO) framework categorizes and annotates cellular components, molecular functions, and biological processes (20). The Kyoto Encyclopedia of Genes and Genomes (KEGG) facilitates the systematic analysis of gene functions, integrating genomic information to investigate potential pathways (21). Gene Set Enrichment Analysis (GSEA) offers statistical insights into the biological significance of gene sets (22, 23). The aforementioned 2869 shared DEGs underwent GO, KEGG enrichment analysis, and GSEA (in GSE57345 and GSE37171, respectively) using the ‘clusterProfiler’ package in R. Pathways for GO and KEGG enrichment were selected based on an adjusted p-value < 0.05 and q-value < 0.05. The top five upregulated and downregulated pathways were visualized using the package ‘ggplot2’ in R. Furthermore, the top 10 hub genes were enriched pathways using Reactome website (https://reactome.org/) and also performed the GO and KEGG analysis. Finally, the signature genes were set as median symbol and performed the GSEA in GSE57345 and GSE37171, respectively.

### Protein-protein interaction network and hub genes selection

In order to streamline the genes selection for protein-protein interaction (PPI) network analysis, 543 genes were filtered from the 2869 shared genes based on a stringent threshold of |logFC| > 0.2 and adjusted p-value <0.05. These genes were next submitted to the STRING database (http://string-db.org/) to compile the interactions of the target proteins, setting a medium confidence score threshold of 0.4. The Cytoscape software (version 3.10.1) was utilized to construct a comprehensive PPI network, judiciously excluding any proteins that lacked connections. Furthermore, the hub genes were selected using the ‘cytoHubba’ plug-in within Cytoscape.

### Machine learning and signature genes selection

Three machine learning algorithms, including Random Forest (RF), Support Vector Machine Recursive Feature Elimination (SVM-RFE), and Least Absolute Shrinkage and Selection Operator (LASSO), were employed to refine the selection of signature genes. This process leveraged the top 10 hub genes previously identified, aiming to bolster classification performance. RF, known for its proficiency in predicting stable factors through an ensemble of decision trees, thus enhancing prediction accuracy, was implemented using the ‘randomForest’ package in R (24). SVM-RFE, distinguished for pinpointing the most discriminative gene set to ensure robust feature selection, was executed via the ‘e1071’ package in R, with the selection criterion in minimal cross-validation error (25). LASSO, a regression algorithm acclaimed for its variable selection efficiency by minimizing classification error probability, here was carried out using the ‘glmnet’ package in R (26). Collectively, the disease-related signature genes were discerned from the overlapping of results across three machine learning for further analysis.

### Expression level and Gene set variation analysis of signature genes

The RNA expression levels of signature genes were obtained from the purified and standardized GSE57345 and GSE37171 datasets, and subjected to a t-test comparison between healthy controls and HF or KF patients. The results were visualized using violin plots in R. Gene Set Variation Analysis (GSVA) is a non-parametric, unsupervised method for evaluating gene set enrichment through the transformation of gene expression data. GSVA scores were employed to evaluate the biological activity of the signature genes using the ‘GSVA’ package and visualized through box plots in R.

### Receiver operating characteristic curves

To assess the correlation between signature genes and disease outcomes, Receiver Operating Characteristic (ROC) curves were employed. Utilizing the ‘pROC’ package in R, ROC curves were plotted, and the area under the curve (AUC) was calculated to determine the predictive power of the signature genes. This analysis was conducted on two training datasets, GSE57345 for HF and GSE37171 for KF, and corroborated with on two validation datasets, GSE135055 for HF and GSE97709 for KF. The resulting AUC values provided a robust measure of the genes’ discriminative capacity in relation to the disease states under investigation.

### Development and validation of the nomogram

A nomogram model was developed utilizing multivariate logistic regression analysis, incorporating the two signature genes to diagnose HF and KF, performing with the ‘rms’ package in R. “Points” refers to each predictor’ score, while the “total points” aggregate these scores, reflecting the cumulative assessment value derived from each predictor. The nomogram’s predictive accuracy was evaluated using a calibration curve, and its discriminative ability was quantified by the ROC curve.

### Molecular docking analysis of bifunctional compounds

To develop a bifunctional compound that activates the CDK2 enzyme while inhibiting CCND1, potential activators of CDK2 and CCND1 inhibitor were screened from the ChEMBL (https://www.ebi.ac.uk/chembl) database. Out of 22,144 assay data entries, only three compounds (CID141497232, CID170906997, CID31703) showed induction of CDK2 expression increase. The 3D structural conformers of these CDK2 activators and the CCND1 inhibitor (CID23424592) were downloaded in SDF file format from the PubChem (https://pubchem.ncbi.nlm.nih.gov) database. An inhibitor of CDK2 (CID23667627) and an activator of CCND1 (CID137657657) were also screened and downloaded. These conformers were converted to PDB format using the Open Babel GUI (https://openbabel.org/docs/GUI/GUI.html) and later to PDBQT format using AutoDockTools-1.5.7 (http://autodock.scripps.edu/resources/adt). The crystal structures of CCND1 (PDB ID: 2W96) and CDK2 (PDB ID: 2A4L) were obtained from the Protein Data Bank (PDB) (https://www.rcsb.org) and eventually converted to PDBQT format using AutoDockTools. Molecular docking was then performed using AutoDockTools-1.5.7 to assess the binding interactions between the selected compounds and their respective protein targets. The docking results were visualized using the PyMOL Molecular Visualization System 2020.

### Immune infiltration and correlation analysis

To delineate the immune cell landscape within HF, KF, and control groups, we employed CIBERSORT (https://cibersort.stanford.edu/), an advanced computational tool that quantifies the abundance of diverse immune cell types. This analysis was conducted using the LM22 signature matrix for datasets GSE57345 and GSE37171. Subsequently, Pearson correlation analysis was applied to discern the correlation among the 22 immune cell types and to elucidate their association with the two signature genes across both datasets. The results were visualized through the ‘ggplot2’ package in R.

### Statistical analyses

All statistical analyses were performed using R software. Differences between data from two sample groups were calculated using an unpaired, two-tailed t-test. Pearson or Spearman correlation coefficients were used to assess the correlation between variables. Differences were considered significant at p < 0.05 (*p < 0.05, **p < 0.01, ***p < 0.001, ****p < 0.0001).

## Results

### Identification of DEGs in HF and KF and their functional enrichment analysis

**Figure 2:**
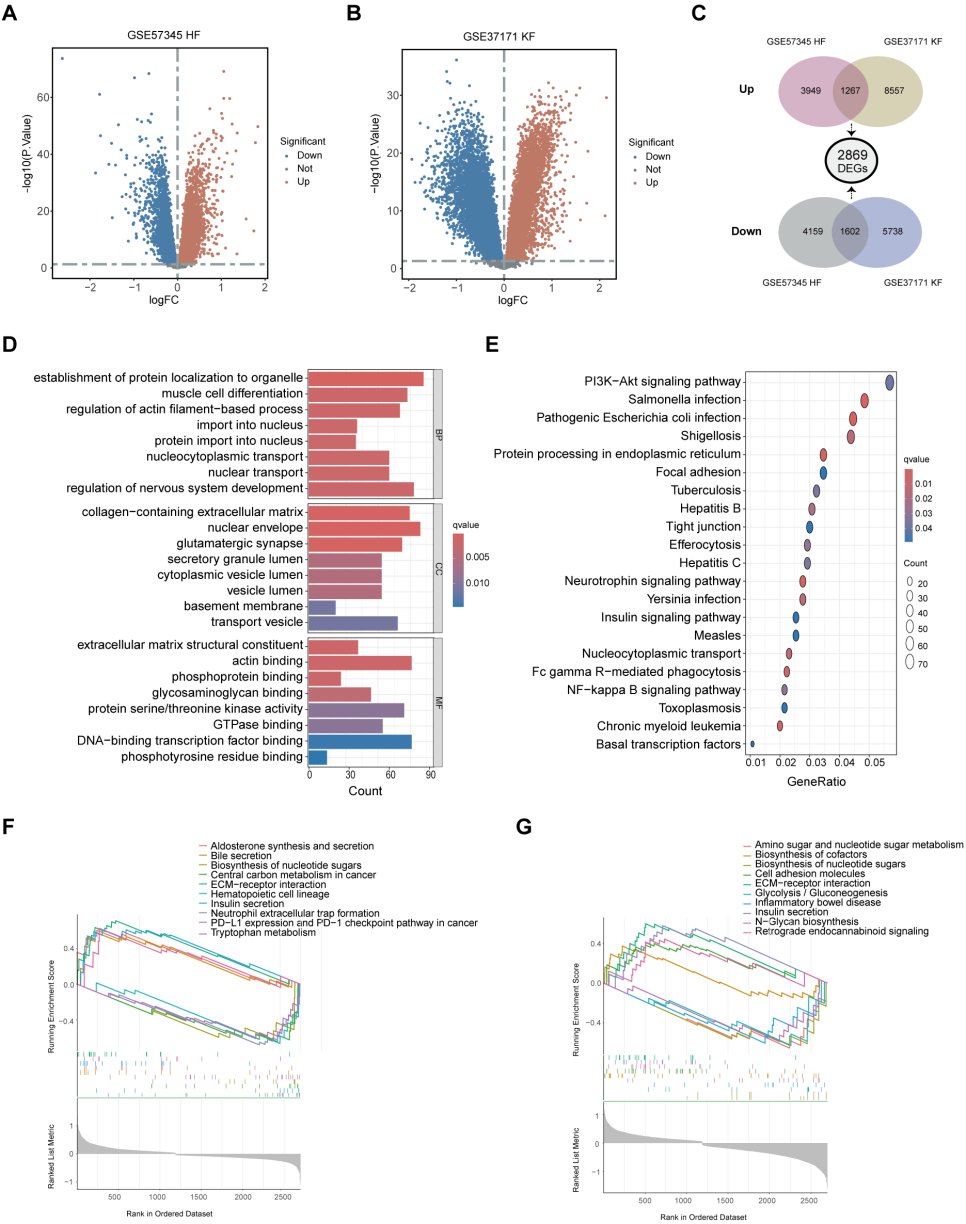
Identification of shared DEGs and their functional enrichment analysis. **(A)** The volcano plot illustrating DEGs between HF patients and healthy controls in GSE57345. **(B)** The volcano plot showcasing DEGs between KF patients and healthy controls in GSE37171. **(C)** A total of 2869 shared DEGs were identified, comprising 1602 downregulated and 1267 upregulated common DEGs from the overlaps of GSE57345 and GSE37171. **(D)** Shared DEGs were enriched through GO terms in BP, CC, and MF. **(E)** Shared DEGs were further enriched through KEGG pathway approach. **(F, G)** GSEA was performed on shared DEGs in GSE57345 and GSE37171, respectively. DEGs, differently expressed genes; HF, heart failure; KF, kidney failure; GO, Gene Ontology; BP, biological process; CC, cellular component; MF, molecular function; KEGG, Kyoto Encyclopedia of Genes and Genomes; GSEA, Gene Set Enrichment Analysis.

To investigate the molecular interplay between cardiac disease and renal dysfunction, we analyzed the microarray dataset GSE57345 for heart failure (HF) and GSE37171 for kidney failure (KF), both sourced from GEO database. DEGs were initially identified by comparing HF patients with healthy individuals, and KF patients with healthy individuals respectively using the ‘limma’ package in R. The significance thresholds set at an adjust p-value < 0.05 and |log2FC| > 0 for ensuring that no biologically significant DEGs are overlooked. The resulting volcano plots (**Figures 2A, B**) graphically represent the DEGs identified. A Venn diagram subsequently revealed a total of 2869 shared DEGs, with 1602 genes downregulated and 1267 genes upregulated across both HF and KF conditions (**Figure 2C**). Next, functional enrichment analysis for these DEGs were performed based on R language. GO analysis indicated that the 2869 DEGs were predominantly associated with processes such as “establishment of protein localization to organelle”, “muscle cell differentiation”, “regulation of actin filament-based process”, and “regulation of nervous system development” in biological process (BP). In the cellular component (CC) ontology, shared DEGs were primarily localized to “collagen-containing extracellular matrix (ECM),” “nuclear envelope,” and “transport vesicle”. For molecular function (MF), significant contributions were noted in “actin binding”, “protein serine/threonine kinase activity”, and “DNA-binding transcription factor binding”. These findings suggest a role for the shared DEGs in maintaining cell structure, cellular homeostasis, cell communication, ECM production and fundamental cell growth (**Figure 2D**). KEGG analysis highlighted that the shared DEGs were significantly enriched in the “PI3K-Akt signaling pathway” (**Figure 2E**). GSEA was conducted on both disease patients and healthy controls to gain deeper insights into the biological signaling of DEGs. The enriched terms, consisting the top 5 upregulated and top 5 downregulated pathways, are illustrated in **Figures 2F, G** for GSE57345 and GSE37171, respectively. These pathways are linked to cellular biochemical reactions, cellular replication capabilities, fibrosis, and inflammation. Additionally, pathways such as “biosynthesis of nucleotide sugars,” “ECM-receptor interaction,” and “insulin secretion” were found to be enriched in either HF or KF through GSEA.

### Unveiling common molecular signatures in HF and KF via PPI network and hub genes

**Figure 3:**
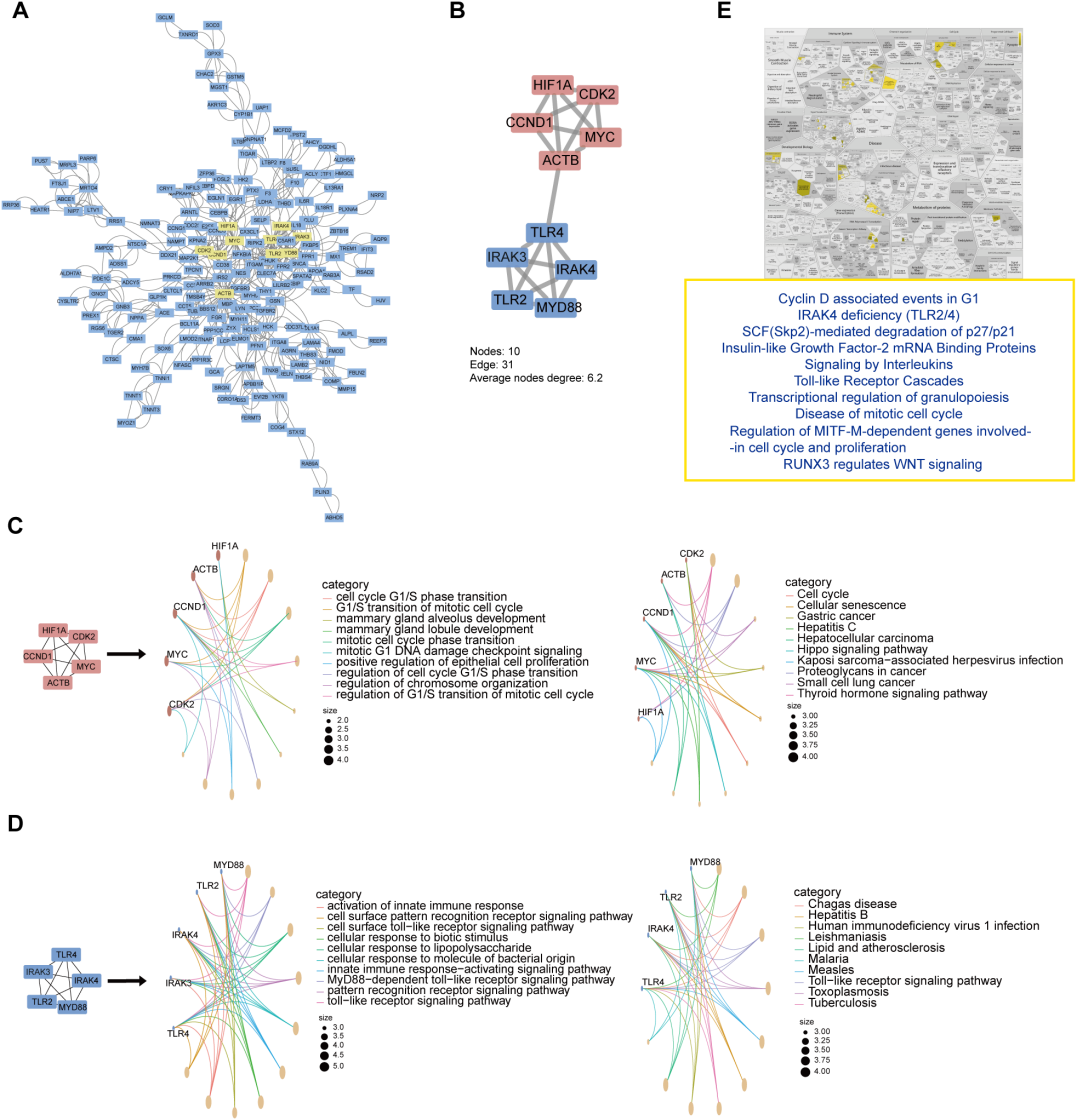
PPI networks, modular analysis of shared DEGs, and functional enrichment of top 10 hub genes. **(A)** The PPI network of selected DEGs, with the top 10 hub genes indicated in yellow indicates. **(B)** Top 10 genes clustering modules were screened via Cytohubba plugins. **(C, D)**. GO and KEGG enrichment analysis of upper red circle genes and lower blue circle, respectively. **(E)** Reactome pathway analysis of top 10 hub gene. DEGs, differentially expressed genes; GO, Gene Ontology; KEGG, Kyoto Encyclopedia of Genes and Genomes.

In the pursuit of elucidating common molecular signatures between HF and KF, DEGs with an adjusted p value < 0.05 and |log2FC| > 0.2 were selected to underscore the intricate protein interactions between HF and KF. We obtained a pronounced PPI network comprising 191 nodes and 901 interactions (**Figure 3A**), after exclusion of unconnected and weakly connected nodes. Key nodes and subnetworks were further established through the Cytohubba plugins, leading to the selection of the top ten hub genes: HIF1A, CDK2, MYC, CCND1, ACTB, TLR4, IRAK4, IRAK3, TLR2, and MYD88. These genes were central to two distinctly interconnected protein clusters, depicted in red and blue (**Figure 3B**).

Next, GO and KEGG analysis of these two clusters revealed that the red network chiefly pertains to cell cycle and senescence (**Figure 3C**), while the blue network is mainly associated with the immune response and inflammation (**Figure 3D**). Particularly, the ten hub genes focus on the “Cyclin D associated events in G1” and “Toll-like Receptor Cascades” biological processes via Reactome pathway analysis (**Figure 3E**). These implicate that the disorder of cell cycle and immune system may play pivotal signaling roles, with the interlinks between ACTB and TLR4 potentially driving the common pathogenesis in both heart and kidney failure.

### The role of signature genes CDK2 and CCND1 in HF and KF

**Figure 4:**
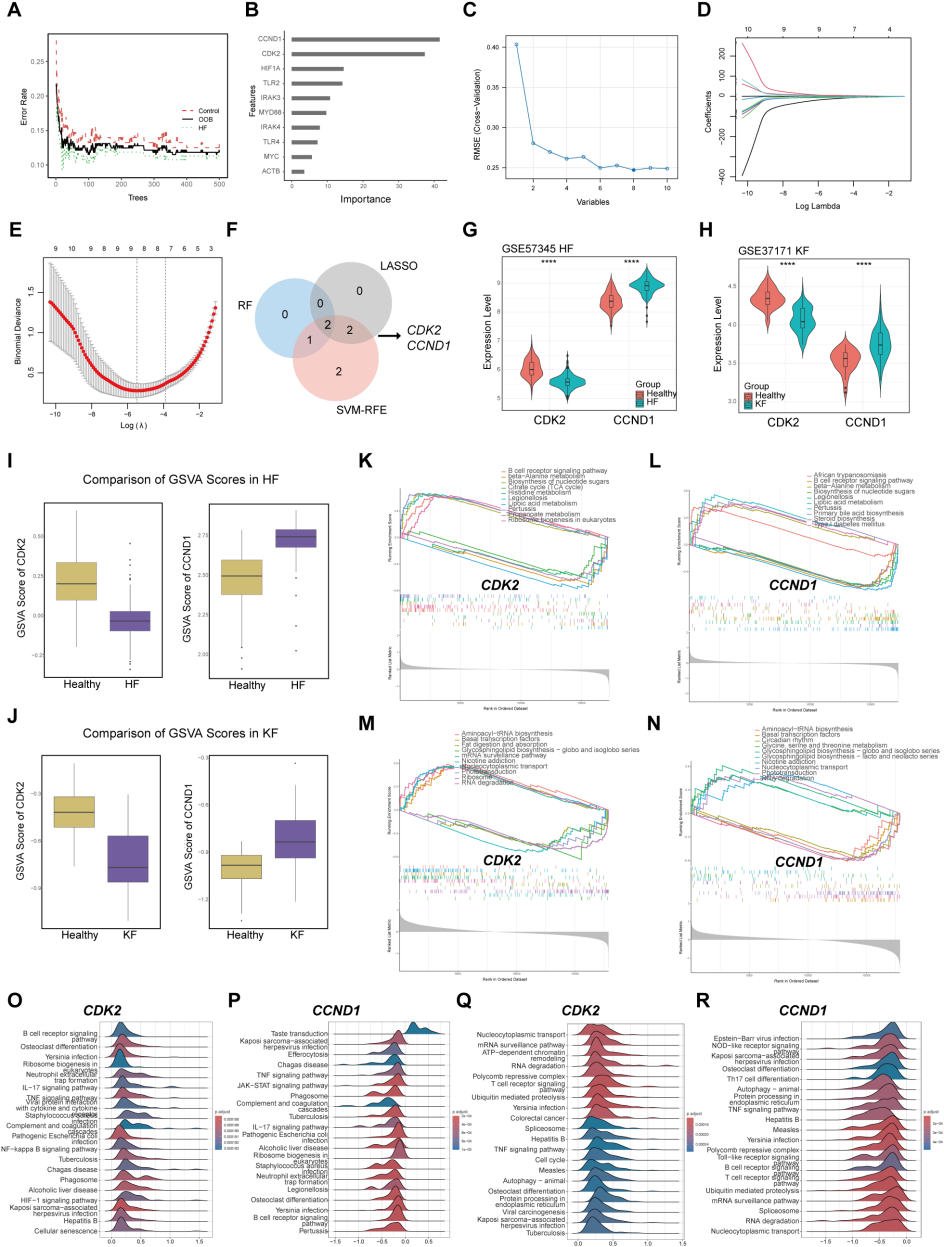
The role of signature genes selected by machine learning methods in HF and KF. **(A, B)** Representative illustrations of the relationship between the number of decision trees and the model error, and the ranking of the top 10 genes based on the importance score in GSE57345. **(C)** Representative image of the RMSE of screening signature genes using the SVM-RFE algorithm for GSE57345. The minimal RMSE is 0.1975 corresponds to eight candidate genes. **(D, E)** Representative images of signature screening in the LASSO model in GSE57345. The minimal point of the curve with n = 8 is the most suitable for GSE57345 based on the 10 hub genes. **(F)** Identification of CDK2 and CCND1 as signature genes by overlapping RF, SVM-RFE, and LASSO using Venn diagram. **(G)** Expression level of CDK2 and CCND1 in GSE57345, **(H)** in GSE37171. ****p<0.0001 by t-test. **(I)** Comparison of GSVA scores of CDK2 and CCND1 in GSE57345, **(J)** in GSE337171. **(K, L)** GSEA for CDK2 and CCND1 in GSE57345, **(M, N)** in GSE37171. **(O, P)** The top 20 enriched pathways by GSEA for CDK2 and CCND1 in GSE57345, **(Q, R)** in GSE37171. HF, heart failure; KF, kidney failure; RMSE, Root Mean Square Error; SVM-RFE, Support Vector Machine-Recursive Feature Elimination. LASSO, Least Absolute Shrinkage and Selection Operator. GSEA, Gene Set Enrichment Analysis; GSVA, Gene Set Variation Analysis.

For the screening of the key genes affecting both diseases, RF, SVM-RFE, and LASSO algorithms were performed to select signature genes based on the 10 hub genes. In the RF algorithms, three feature genes were extracted as significant by overlapping of the top 6 genes from GSE57345 and GSE37171 according to their importance in MeanDecreaseGini value. The RF classifier was setting with optimal number of trees to minimize error rates and maximize stability (**Figure 4A**), and the importance of the 10 hub gens was ranked (**Figure 4B**). The SVM-RFE model identified a total of 7 genes with minimal root-mean-square error (RMSE) values of 0.1185227 in GSE37171 (**Figure 4C**) and 0.2507178 in GSE57345. LASSO regression pinpointed 4 common genes in GSE37171 with a lambda.min value of 0.003800386, and in GES57345 with a lambda.min value of 0.01289927 for next analysis (**Figures 4D, E**). The culmination of this analysis was the identification of CDK2 and CCND1 as the signature markers in HF and KF, as depicted in the Venn diagram (**Figure 4F**). All the results of machine learning for GSE57345 and GSE37171 are listed in **Supplementary Table 2**.

Further analysis revealed a marked decrease of CDK2 expression, while CCND1 levels exhibited a significant increase in both the HF and KF groups compared to the healthy group (**Figures 4G, H**). The GSVA score results indicated that the bioactivity of CDK2 is significantly lower in HF and KF patients compared to controls, whereas CCND1 shows opposite (**Figures 4I, J**). We investigated the signaling pathways enriched by the two signature genes to explore their potential molecular mechanisms affecting the progression of HF and KF. GSEA results demonstrated that CDK2 is associated with the top 5 downregulated and upregulated pathway related to metabolism, infection, and biosynthesis of nucleotide sugars in HF (**Figure 4K**), and genetic regulation, cell signaling and transport in KF (**Figure 4M**). CCND1 shows significant enrichment in inflammation, metabolism, and biosynthesis of nucleotide sugars in HF (**Figure 4L**), and gene transcription, circadian rhythm in KF (**Figure 4N**). The top 20 enriched pathways analyzed by GSEA for the signature genes are listed in **Figures 4O–R**. Here suggests that the signature genes play a significant role in the progression of HF and KF by modulating inflammatory response, cellular metabolism, apoptosis, and cell cycle.

### ROC curves analysis of signature genes in HF and KF

**Figure 5:**
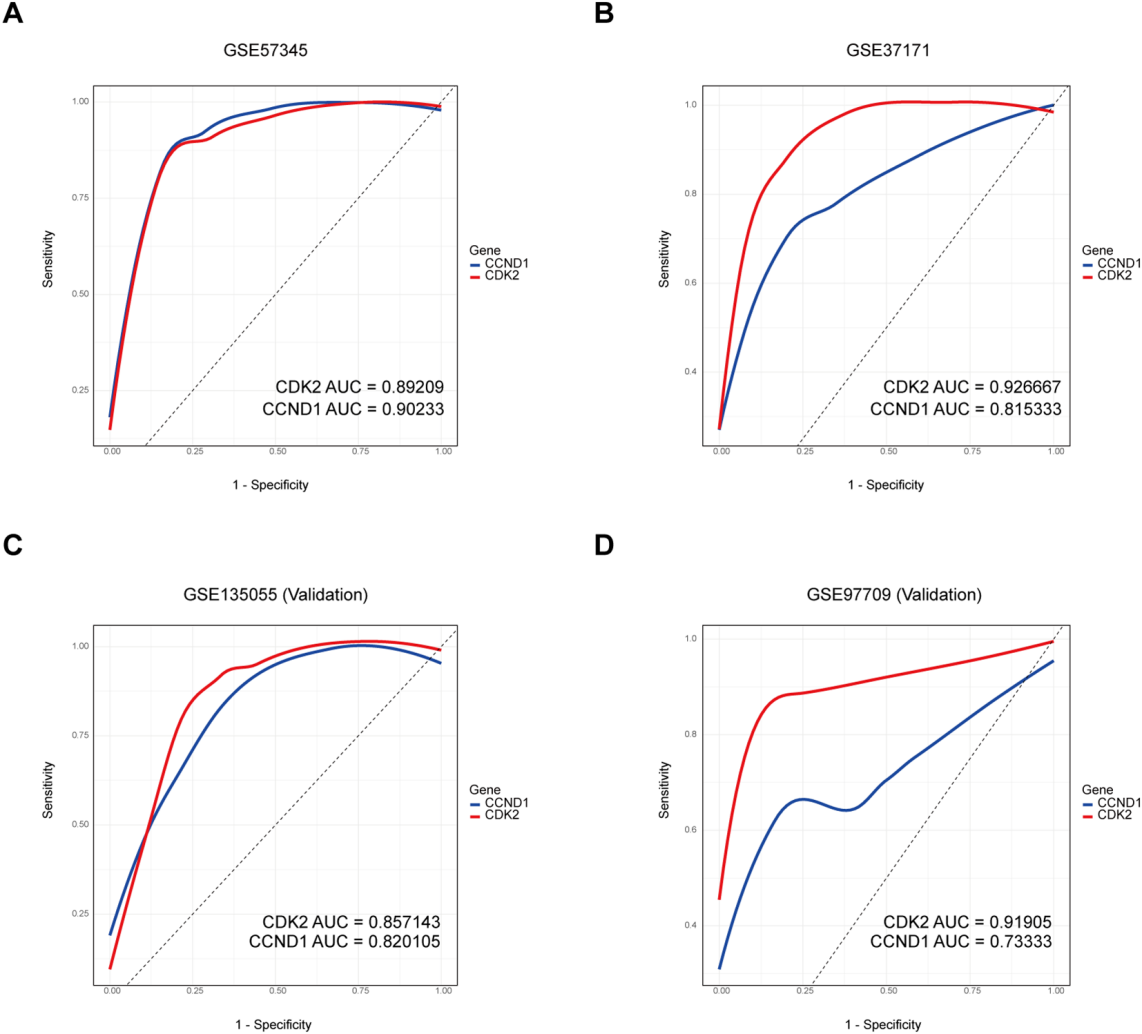
ROC curves of the signature genes in HF and KF. **(A, B)** The ROC curves of CDK2 and CCND1 in the training set containing GSE57345 and GSE47171. **(C, D)** The ROC curves of CDK2 and CCND1 in the validation set including GSE135055 and GSE97709. ROC, Receiver Operating Characteristic; HF, heart failure; KF, kidney failure.

ROC curve analysis was utilized to investigate the diagnostic potential of the key genes CDK2 and CCND1 across training datasets GSE57345 for HF and GSE37171 for KF, as well as validation datasets GSE135055 for HF and GSE97709 for KF. The AUCs for CDK2 were 0.89209 in HF and 0.926667 in KF, for CCND1, they were 0.90233 in HF and 0.815333 in KF. These ROC curves demonstrated the genes’ exceptional ability to differentiate between HF and KF patients from healthy individuals (**Figures 5A, B**). Furthermore, the predictive value of CDK2 and CCND1 as biomarkers was validated with AUCs of 0.857143 and 0.820105 in GSE135055, and 0.91905 and 0.73333 in GSE97709, respectively (**Figures 5C, D**). The results implicate the significant roles that CDK2 and CCND1 may play in the development of both HF and KF.

### Nomogram assessment and validation of diagnostic markers

**Figure 6:**
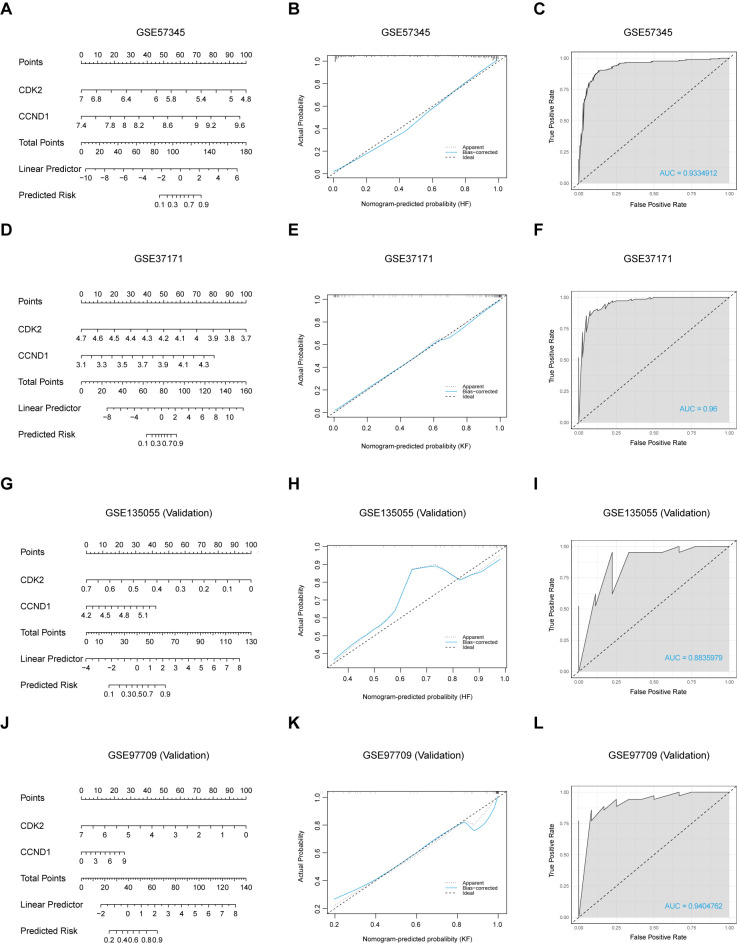
Construction of a nomogram and evaluation of its diagnostic value. **(A, D, G, J)** Visible nomograms for diagnosing HF and KF in training sets GSE57345 and GSE37171, and validation sets GSE135055 and GSE97709, respectively. **(B, E, H, K)** Calibration of the nomogram model in the respective datasets. **(C, F, I, L)** ROC plot assessments of the predictive model for each dataset.

A diagnostic nomogram model was constructed on the two signature genes, CDK2 and CCND1, to predict the likelihood of HF and KF occurrences via either training set or validation set (**Figures 6A, D, G, J**), resulting in a high level of precision in forecasting heart and kidney failure risk. The nomogram’s predictive accuracy was initially assessed using calibration curves for the four datasets, GSE57345, GSE37171, GSE135055, and GSE97709 (**Figures 6B, E, H, K**). Subsequently, ROC analysis was developed to evaluate the precision and stability of the nomogram model across the four datasets, with AUCs of respective 0.9334912, 0.96, 0.8835979 and 0.9404762, for GSE57345, GSE37171, GSE135055 and GSE97709 (**Figures 6C, F, I, L**). Both signature genes, CDK2 and CCND1, possess a potential clinical value in effectively distinguishing HF and KF patients from healthy controls, offering promising diagnostic biomarkers for cardiorenal disease.

### Evaluation of bifunctional CDK2/CCND1 modulators for candidate drug development

With the aim of targeting the signature genes CDK2 and CCND1 for potential therapy in HF and KF, an initial screening was conducted via the ChEMBL database (https://www.ebi.ac.uk/chembl/) to identify compounds that could simultaneously activate the CDK2 enzyme and inhibit CCND1. However, this screening did not reveal any common chemical entities, and only three compounds were identified as capable of activating CDK2.

**Figure 7:**
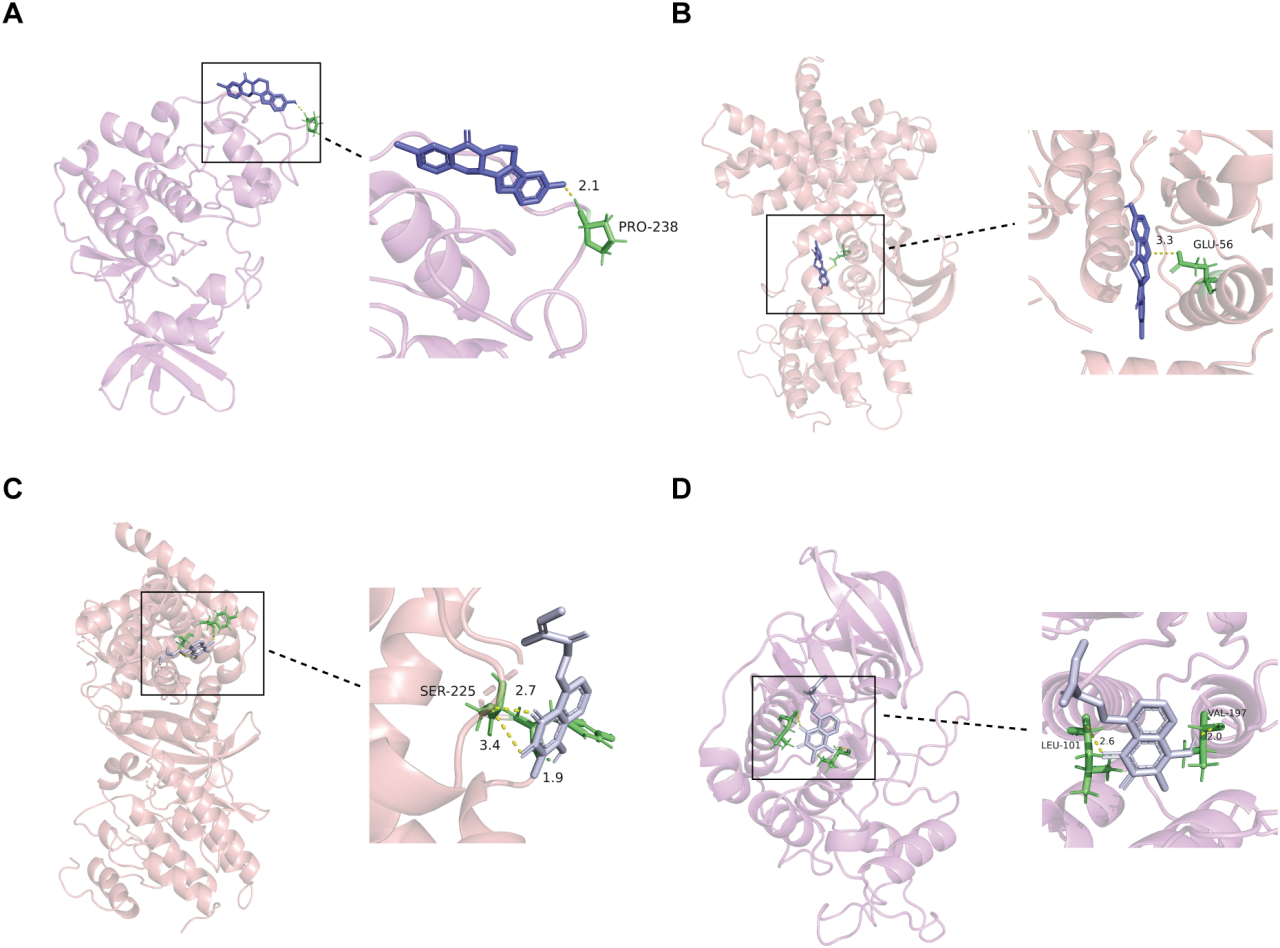
Interaction between selected ligands and CDK2 or CCND1. **(A)** 3D docking of CDK2 activator (CID14149723) into the CDK2 active site. **(B)** 3D docking of CDK2 activator (CID14149723) into the CCND1 active site. **(C)** 3D docking of CCND1 inhibitor (CID23424592) into the CCND1 active site. **(D)** 3D docking of CDK2 activator (CID23424592) into the CDK2 active site. CID14149723, a known activator of CDK2; CID23424592, a known inhibitor of CCND1.

Investigating protein-drug interactions through molecular docking is crucial for understanding binding dynamics and potential therapeutic roles (27). In this study, the three potential CDK2 activators were subjected to molecular docking with the CDK2 and CCND1 proteins, with the resulting binding energies presented in **Supplementary Table 3**. Compound CID141497232 exhibited the lowest binding energies of -5.63 kcal/mol with CDK2 and -5.55 kcal/mol with CCND1, indicating the most stable docking outcomes (**Figures 7A, B**). Additionally, docking analysis was performed using Compound CID23434592, a known CCND1 inhibitor, which showed binding energies of -4.04 kcal/mol with CCND1 and -3.89 kcal/mol with CDK2 (**Figures 7C, D**). Negative control docking was also conducted using CID23667627, a known CDK2 inhibitor, and CID137657657, a known CCND1 activator, resulting in binding energies of -5.19 kcal/mol and -6.03 kcal/mol, respectively (**Supplementary Figures 2A, B**). In terms of binding interactions, CID141497232 (the CDK2 activator) formed a hydrogen bond with the Glu-56 residue of CCND1, whereas CID23424592 (the CCND1 inhibitor) formed two hydrogen bonds with Ser-225. CID137657657 (the CCND1 activator) interacted with Val-96, His-95, Asp-158, and Ala-16, forming hydrogen bonds with each. Moreover, CID23424592 also bound to CDK2 at Leu-101 and Val-197, while CID141497232 formed a hydrogen bond with Pro-238. In contrast, CID23667627 (the CDK2 inhibitor) interacted with Lys-250, forming a hydrogen bond.

While binding energies and binding modes provide insights into the strength and stability of these interactions, they do not directly determine whether a compound will function as an activator or inhibitor. To conclusively establish the functional roles of these compounds, further studies involving biological activity assays are necessary.

### Immune cell infiltration and correlation with signature genes

Given our observation that the 10 hub genes from the shared DEGs of HF and KF were strongly enriched in immune and inflammation pathway (**Figure 3D**), and considering that the signature genes CDK2 and CCND1 emerged as potential diagnostic biomarkers for both conditions via nomogram model, we performed immune cell infiltration analysis to delve deeper into the immune regulation of HF and KF.

**Figure 8:**
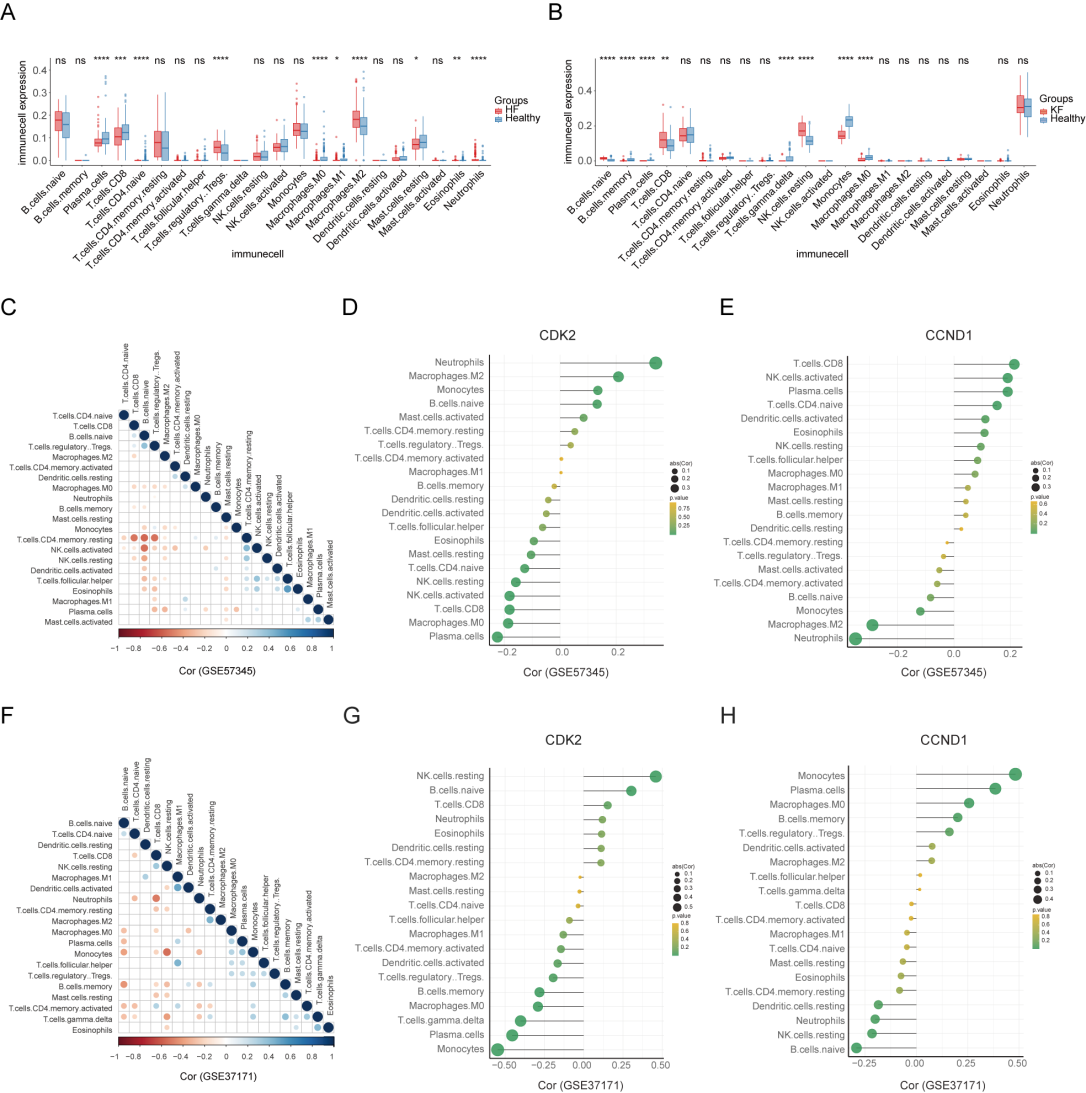
Landscape of immune cell infiltration and their correlation with the signature genes. **(A, B)** The distribution of 22 immune cell types between HF and KF patients and healthy controls in datasets GSE57345 and GSE37171, respectively. **(C, F)** The correlation of diverse immunocytes in HF and KF samples in datasets GSE57345 and GSE37171, respectively. **(D, E, G, H)** Lollipop charts showing the correlation of the signature genes, CDK2 and CCND1, with immunocytes in HF and KF samples in datasets GSE57345 and GSE37171, respectively. HF, heart failure; KF, kidney failure. *p<0.05, **p<0.01, ***p<0.001 and ****p<0.0001 by t-test.

Employing the CIBERSORTx algorithm, the HF group exhibited a higher proportion of plasma cells, CD8+ T cells, naive CD4+ T cells, M0 macrophages, M1 macrophages, resting mast cells, and eosinophils, and a lower proportion of M2 macrophages and neutrophils compared to the healthy group, across the spectrum of 22 immune cell types (**Figure 8A**). Conversely, the KF group showed a significant increase in memory B cells, plasma cells, gamma delta T cells, monocytes, and M0 macrophages, and a decrease in naive B cells, CD8+ T cells, and resting NK cells relative to controls (**Figure 8B**). Correlation analysis among the 22 immune cell types revealed that resting memory CD4+ T cells were negatively correlated with CD8+ T cells, naive B cells, and regulatory T cells, as well as activated NK cells with naive B cells in GSE57345 (**Figure 8C**). Additionally, neutrophils and monocytes demonstrated a negative association with CD8+ T cells and resting NK cells in GSE37171 (**Figure 8F**).

Further investigation into the role of signature genes in immune response was conducted through a correlation analysis between signature genes and immune cells (**Figures 8D, E, G, H**). CDK2 and CCND1 were found to have significant associations with multiple immune cells, particularly neutrophils, monocytes, and resting NK cells. Consequently, we propose that CDK2 and CCND1 may play a crucial role in immune response, potentially through signaling pathways involving neutrophils, monocytes, and resting NK cells, which are related to the pathophysiology of both heart and kidney failure.

## Discussion

Clinical evidence indicates a high prevalence of cardiac and renal co-dysfunction among numerous patients, due to the intricate relationship between the heart and kidney, contributing to the rising global mortality rate (28). Unraveling the underlying mechanism responsible for the interaction between cardiovascular diseases and kidney disease could illuminate innovative therapies for disorders of both heart and kidneys, further establishing beneficial treatment for cardiorenal syndrome. This investigation delves into the molecular signatures of shared pathways in both heart failure and kidney failure using bioinformatics analysis, and identifies two novel biomarkers based on machine learning related to the diagnosis and cure of both HF and KF.

In this study, we initiated our analysis by acquiring two datasets from GEO: GSE57345, patients from the diagnosis of heart failure (HF), and GSE37171, patients concerning the criteria for uremia, herein referred to as kidney failure (KF). A total of 2869 DEGs were identified, including 1602 downregulated and 1267 upregulated, shared between HF and KF. These DEGs were annotated through GO, KEGG, and GSEA functional enrichment analysis, revealing a significant association with the PI3K-Akt signaling pathway, inflammatory response, metabolic process, and extracellular matrix organization. The PI3K-Akt pathway, integral to cellular functions such as cycle regulation, proliferation, metabolism, survival, growth, and angiogenesis, is deemed critical in the pathogenesis of HF and KF (29, 30). To further elucidate the principal molecular clusters common to HF and KF, PPI analysis was conducted, unveiling hub genes within two protein clusters linked by an interaction edge through the CytoHubba plug-in of Cytoscape. Remarkably, these clusters exhibit strong associations with cell cycle and inflammation by GO, KEGG and Reactome enrichment analysis, suggesting that dysregulation in cell cycle and inflammation mechanisms may drive the pathogenesis of both heart and kidney disorders. Such insights could provide a creative understanding of the therapeutic approaches for HF, CKD, and cardiorenal syndrome.

The cell cycle’s role in disease pathophysiology is well-documented (31). Its dysregulation can lead to pathological changes in cardiac and renal tissues, exacerbating HF and CKD. Post-cardiac injury, the disruption of cell cycle regulation triggers apoptotic pathways, hindering cardiomyocyte regeneration due to the heart’s limited regenerative capacity, thus potentially precipitating HF (32–34). Similar mechanisms in the kidneys, where renal tubular epithelial cells may succumb to apoptosis following cell cycle arrest after injury (35). Non-functional scar tissue with the production of extracellular matrix proteins replaces the lost cardiomyocytes in heart, also the arrest at the G2/M phase of cell cycle enhances the secretion of profibrotic factors in kidney, together as a result in fibrosis and failing organs (9, 36). Furthermore, recent reports suggest that cell cycle dysregulation is relative to oxidative stress, extracellular matrix, and the immune system (37).

Inflammation also plays a critical role in the progression of HF and CKD, involving a multitude of cytokines, chemokines, and immune system components (38). In both diseases, inflammation is not merely a consequence but also a potent drive. The inflammation response in HF and CKD is characterized by the upregulation of pro-inflammatory mediators like tumor necrosis factor-α (TNF-α), interleukin-1β (IL-1β), and interleukin-6 (IL-6) in the tissue and bloodstream, which are produced in response to neurohormonal and sympathetic triggers (39). Furthermore, this leads to exacerbated myocardial inflammation and fibrosis, while in CKD, it contributes to tubular damage and worsened renal function (9, 40). Our findings indicate a significant enrichment of genes associated with the Toll-like receptor pathway within our identified blue PPI network cluster, underscoring its pivotal role in the inflammatory processes of HF and CKD.

The advent of artificial intelligence (AI) and machine learning has revolutionized the processing of biological data, offering fresh insights and approaches for biological research. In our study, we combined the machine learning of RF, SVM-RFE, and LASSO to screen the signature genes based on the expression profiles of the top 10 hub genes identified. The culmination of this integrative machine learning strategy was the discovery of CDK2 and CCND1 as novel biomarkers for both HF and KF. The GSVA score and GSEA analysis for CDK2 and CCND1 revealed the signaling pathways potentially modulating the progression of HF and KF. The diagnostic relevance of these biomarkers for HF and KF was corroborated across the initial training set and subsequent validation set.

Additionally, a molecular docking study was performed to assess interactions between CDK2 and CCND1 with three CDK2-activating ligands. A selected CCND1 inhibitor was also docked with both CDK2 and CCND1. This analysis elucidates the molecular mechanisms of these proteins and provides a structural basis for further drug design efforts. To validate their roles as inhibitors or activators, further cytotoxicity testing is essential. The design of polypharmacological drugs poses significant challenges, particularly due to the limited number of known CDK2 activators. Nonetheless, Chen et al. utilized chemoinformatics to identify conformationally flexible scaffolds capable of adapting to both activation and antagonism-related pockets (41). However, their approach requires a substantial number of activator scaffolds, underscoring the importance of discovering more CDK2 activators to enhance the existing database.

CDK2 (cyclin-dependent kinases 2) gene, a catalytic subunit of the cyclin-dependent kinase (CDK) complex, controls the G1 to S phase transition by binding with cyclins, where it is essential for DNA replication and cellular entry into the S phase (42). It is known that CDK2 has significant implications for cancer treatment and cellular aging (43). In HF and CKD, Su et al. found an association between CDK2 and impaired autophagic flux, which mitigated the cardiac remodeling in mice with heart failure (44). Cedric et al. observed that mice deficient in CDK4 and CDK2 are able to complete embryonic progression but die shortly after birth due to heart failure (45). Meanwhile, Turgay et al. reported that the CDK2 inhibitor roscovitine aggravated renal injury and fails to prevent the tubulointerstitial fibrosis by Cul3 deletion (46). Also, enhancing the cyclin-D1/cyclin-E2/CDK2/CDK4 axis has been shown to maintain renal function in CKD models (47). These findings corroborate our data, revealing a strong inverse correlation between CDK2 expression and the onset of HF and KF, as illustrated by our predictive diagnostic mode. Although evidence on CDK2’s role in HF and KF is limited, it may significantly influence cell proliferation, apoptosis, senescence, and repair mechanisms, aligning with our PPI results. CCND1 (cyclin D1), another cyclin family member identified in our study, emerged as a promising therapeutic target for HF (48). Its overexpression may contribute to idiopathic dilated cardiomyopathy pathogenesis (49). CCND1 modulates cyclin-dependent kinases (CDKs) by binding with CDK4 or CDK6, thereby facilitating the G1/S cell cycle transition (50), a process which may critically impact HF and KF pathogenesis by regulating the cell cycle in concert with CDK2.

Our PPI result and other studies underscores the importance of inflammation and immune processes in HF and CKD (51). Here, we explored immune infiltration in HF and KF compared to healthy controls using CIBERSORTx, respectively. Our findings indicate a pronounced prevalence of plasma cells and M0 macrophages in both HF and KF, highlighting their critical functions in the immune response. Correlation analyses of immune cells was performed to measure the immune microenvironment in HF and KF. Further exploration into the interplay between immune responses and two signature genes revealed that CDK2 exhibits a negative correlation with plasma cells and M0 macrophages, whereas CCND1 demonstrates the converse. These insights lead us to hypothesize that diminished CDK2 and elevated CCND1 expression may potentiate the immune response by upregulating plasma cells and M0 macrophages in both HF and KF. Furthermore, growing evidence showed a close relationship between cell cycle regulation and the immune system (52, 53), proposing that these two biological processes, associated with CDK2 and CCND1, may synergistically orchestrate the pathophysiology of HF and CKD.

Nevertheless, several limitations of our study should be acknowledged. Firstly, the datasets employed were limited to patients diagnosed with either HF or KF, instead of those with concurrent diagnoses or cardiorenal syndrome due to such datasets are not available in the public database. This delineation may narrow the applicability of our findings, thereby highlighting the imperative for future research including such datasets. Moreover, our approach for selecting the top 10 hub genes from all the hub gene pool is subjective and may inadvertently neglect other hub genes of potential significance in disease progression. Lastly, our findings are predicated on bioinformatics and machine learning analysis. Future validation should include *in vitro* and *in vivo* experiments to further investigate the shared pathogenic mechanisms of HF and KF related to CDK2 and CCND1.

## Conclusion

In conclusion, our research marks for the first exploration into the utilization of an integrated bioinformatics and machine learning framework to analyze transcriptomic data pertinent to HF and KF. We have identified two novel biomarkers, CDK2 and CCND1, and unveiled a significant discovery regarding the interplay between cell cycle regulation and immune response, which may illuminate the shared molecular mechanisms underlying both HF and KF. Our findings underscore the diagnostic relevance of CDK2 and CCND1 in HF and KF, providing potential new insights and targets for the diagnosis and treatment of both conditions, or cardiorenal syndrome. While our research is in its early stages, it lays a robust foundation for further investigation into this vital domain, with the ultimate goal of improving patient care and outcomes.

## Data Availability

The original contributions presented in the study are included in the article/**Supplementary Material**. Further inquiries can be directed to the corresponding author.
