# Supplementary material for: Identification of novel biomarkers, shared molecular signatures and immune cell infiltration in heart and kidney failure by transcriptomics
Source: Front Immunol. 2024 Sep 16;15:1456083. doi: 10.3389/fimmu.2024.1456083 (PMC11439679; doi:10.3389/fimmu.2024.1456083)
Supplement: Supplementary file 1 [file DataSheet1.docx]

**Supplementary table 1**. Datasets and sample information from GEO.

| No. | GSE number | Platform | Diagnosis | Source type | Samples | Group |
| --- | --- | --- | --- | --- | --- | --- |
| 1 | GSE57345 | GPL9052 | Heart failure | Heart tissue | 177 patients and 136 controls | Training |
| 2 | GSE37171 | GPL579 | Uremia | Whole blood | 75 patients and 40 controls | Training |
| 3 | GSE135055 | GPL16791 | Heart failure | Heart tissue | 21 patients and 9 controls | Validation |
| 4 | GSE97709 | GPL17303 | End-stage renal disease | Plasma | 25 patients and 12 controls | Validation |

**Supplementary table 2**. Results of three machine learning methos for HF (GSE57345) and KF (GSE37171) based on the top 10 hub genes.

| Group | RF (top 60%) | SVM-RFE (the minimum of RMSE) | LASSO |
| --- | --- | --- | --- |
| HF (GSE57345) | CCND1, CDK2, HIF1A, TLR2, IRAK3, MYD88 | CCND1, CDK2, HIF1A, TLR2, MYD88, IRAK4, MYC, IRAK3, TLR4 | CCND1, CDK2, IRAK3, HIF1A, MYC |
| KF (GSE37171) | ACTB, CDK2, TLR4, HIF1A, MYC, CCND1 | CDK2, ACTB, TLR4, MYC, HIF1A, CCND1, IRAK4, IRAK3 | ACTB, CCND1, CDK2, IRAK3, IRAK4, MYC, TLR4 |
| Overlap | CCND1, CDK2, HIF1A | CCND1, MYC, CDK2, IRAK3, IRAK4, TLR4, HIF1A | CCND1, MYC, CDK2, IRAK3 |

**Supplementary table 3**. The binding energy for three potential CDK2 activators docking with CDK2 and CCND1, respectively.

| Protein | CID141497232 | CID170906997 | CID31703 | CID23434592 |
| --- | --- | --- | --- | --- |
| CDK2 (PDB ID:2A4L) | -5.63 Kcal/mol | -4.47 Kcal/mol | -2.45 Kcal/mol | -4.04 kcal/mol |
| CCND1 (PDB ID: 2W96) | -5.55 Kcal/mol | -2.94 Kcal/mol | -3.47 Kcal/mol | -3.89 kcal/mol |

Supplementary figure legends:

**Supplementary Figure 1**. Limma analysis of HF and KF patients versus healthy controls. **A-B.** Visualization of sample normalization in HF and KF datasets using Box-and-Whisker Plot. **C-D.** Heatmaps displaying the expression levels of top 50 upregulated and downregulated DEGs identified in the HF and KF datasets, respectively.

**Supplementary Figure 2.** Interaction between selected ligands and CDK2 or CCND1 as negative controls. **A.** 3D docking of CDK2 inhibitor (CID23667627) into the CDK2 active site. **B.** 3D docking of CCND1 activator (CID137657657) into the CCND1 active site. CID23667627, a known inhibitor of CDK2; CID137657657, a known activator of CCND1.
