# Supplementary figures and images for: Identification of novel biomarkers, shared molecular signatures and immune cell infiltration in heart and kidney failure by transcriptomics
Source: Front Immunol. 2024 Sep 16;15:1456083. doi: 10.3389/fimmu.2024.1456083 (PMC11439679; doi:10.3389/fimmu.2024.1456083)

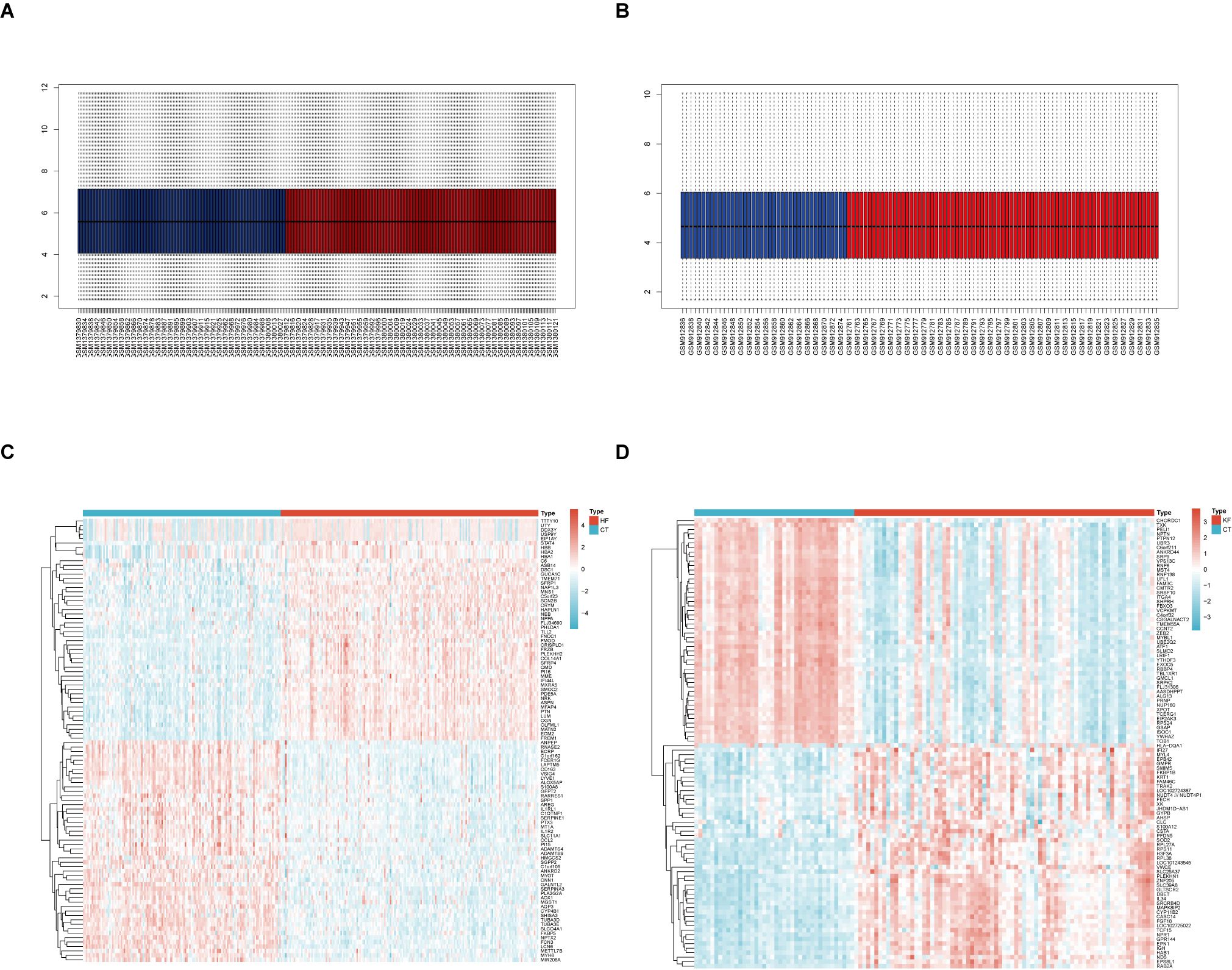

Supplement: Supplementary file 2 [file Image1.tif]

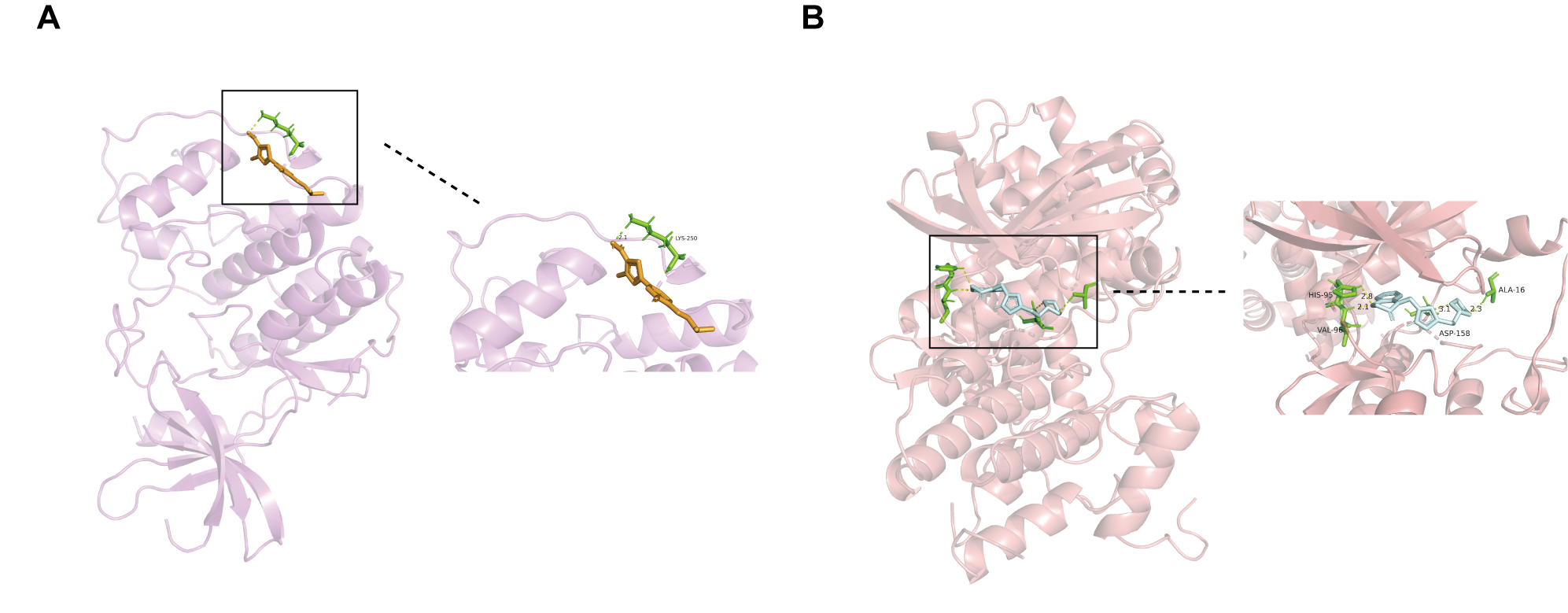

Supplement: Supplementary file 3 [file Image2.tif]
